# Ancient DNA preserved in small bone fragments from the P.W. Lund collection

**DOI:** 10.1002/ece3.7162

**Published:** 2021-02-05

**Authors:** Frederik V. Seersholm, Kasper Lykke Hansen, Matthew Heydenrych, Anders J. Hansen, Michael Bunce, Morten E. Allentoft

**Affiliations:** ^1^ Trace and Environmental DNA (TrEnD) Laboratory School of Molecular and Life Sciences Curtin University Bentley WA Australia; ^2^ Section for GeoGenetics GLOBE Institute University of Copenhagen Copenhagen Denmark; ^3^ Section for Evolutionary Genomics GLOBE Institute University of Copenhagen Copenhagen Denmark; ^4^ Natural History Museum of Denmark University of Copenhagen Copenhagen Denmark

**Keywords:** ancient DNA, bulk bone, metabarcoding, museum collections, palaeontology

## Abstract

The Lund collection is one of the oldest subfossil collections in the world. The vast assemblage of subfossils was collected in the 1830s and 1840s by Peter Wilhelm Lund in Lagoa Santa, Brazil, and was shipped to Copenhagen in 1848, where it was stored in various locations around the city with little attention for the future preservation of the collection. So far, successful genetic research on the material collected by Lund has been limited to two samples of human petrous bone. However, less is known about the preservation conditions of the vast amounts of small and fragmentary bones stored in the collection. To address this, we studied ancient DNA from bulk bone samples of approximately 100 bone fragments from the P.W. Lund collection from boxes with varying degrees of physical preservation conditions. Using bulk bone metabarcoding, we found a high species diversity in all samples. In total, we identified 17 species, representing 11 mammals, two birds, one fish, and three frogs. Of these, two species are new to the collection. Collectively, these results exhibit the potential of future genetic studies on the famous P.W. Lund collection and suggest that the effects of poor storage conditions are probably negligible compared with the long‐term in situ degradation that specimens undergo before excavation.

## Graphical abstract

The Lund collection is one of the oldest fossil collections in the world, but it is not known whether the poorly preserved small mammal bones that constitute the majority of the collection are suitable for ancient DNA analysis. We genetically identify 17 species, representing 11 mammals, two birds, one fish, and three frogs from small fragmentary bones in Lund's collection. These results exhibit the potential of future genetic studies on the famous P.W. Lund collection.

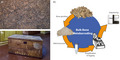

## 1 INTRODUCTION

Museum collections are vital for natural history research. By providing easy access to specimens collected from temporally and spatially diverse locations, comparative collections facilitate the continued progress of research in past ecosystems. In recent years, the study of ancient biomolecules preserved in museum specimens has advanced diverse fields such as paleontology (Allentoft et al., 2014; Barnett et al., 2020), paleoecology (Lorenzen et al., 2011), archeology (Seersholm et al., 2016; Sinding et al., 2017), and anthropology significantly (Moreno‐Mayar et al., 2018; Slon et al., 2018), from large‐scale studies of migration patterns in the past (Allentoft et al., 2015) to studies of ecological changes over tens of thousands of years (Seersholm et al., 2020). However, the successful study of ancient DNA (aDNA) and proteins is greatly dependent on biomolecular preservation. To ensure the continued advancement of natural history on a molecular level, a better assessment of the effects of museum storage on sample preservation is required.

**FIGURE 1:**
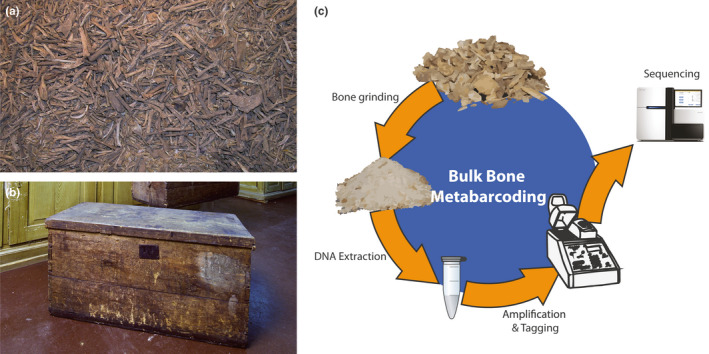
Samples and workflow. (a) Owl regurgitation from Lund's collection. (b) One of the original wooden boxes used to transport the collection from Brazil to Denmark in 1848. (c) Bulk bone metabarcoding workflow

One of the oldest and most famous natural history collections in the world is that of Peter Wilhelm Lund, collected primarily during the years 1835–1845 (Figure 1a,b). P.W. Lund was a Danish naturalist, renowned for his research on subfossil bones from Lagoa Santa in Brazil. His excavations of limestone caves around Lagoa Santa led to the first description of the Brazilian Pleistocene megafauna (Holten & Sterll, 2010), including the South American saber‐toothed cat which he named *Smilodon populator*. Lund also discovered and described numerous species of extinct ground sloths and glyptodonts, as well as approximately 30 early human skeletons. Collectively, his findings from Lagoa Santa laid some of the foundation that would lead Charles Darwin to his theory of evolution (Darwin, 1859).

During his long career, Lund identified more than 800 caves and excavated many tens of thousands of subfossils. Initially, these subfossils were kept in Lund's house in Lagoa Santa, but around 1845, Lund decided to donate his collection to the Danish king Christian the 8th. The subfossils were packed in hundreds of wooden boxes (Figure 1b), which were then carried over land by mule to Rio de Janeiro and shipped to Denmark. Unfortunately for the collection, the king died in 1848 shortly after its arrival in Copenhagen. This led to a tumultuous period where the subfossils were briefly placed on display at Christiansborg Castle and then repacked and moved around between various storage facilities. Finally, in 1858, the collection was absorbed into Copenhagen University's natural history collections and was put on display in 1870 at the new Zoological Museum in Copenhagen. Here, it would remain on permanent display for almost 100 years, only briefly interrupted in 1944, where it was moved to a bomb shelter during the final months of the Second World War (Hansen pers comm). In 1970, the collection was transferred to its current location in Universitetsparken in Copenhagen where it is kept in storage.

Lund's entire collection consists of more than 100,000 bones, as well as more than 2,000,000 small bones from owl regurgitation (Figure 1a) and some 1,300 breccia samples. The material is mainly of late Pleistocene and early Holocene age. With approximately 45 vertebrate type specimens, the collection is today regarded as one of the Natural History Museum of Denmark's (NHMD) finest assets (Hansen, 2020). Of the immense amount of subfossil material that was shipped home, only a fraction was formally catalogued and described by Lund. The majority of the bones in Lund's collection has thus remained in museum storage for around 170 years.

The effect of long‐term museum storage on DNA and proteins is not well understood. While DNA fragmentation is a time‐dependent process (Allentoft et al., 2012), the rate at which this occurs is influenced by environmental factors such as pH, humidity, and the chemical composition of the surroundings. It is therefore impossible to derive a simple correlation between DNA preservation and sample age across different preservation environments (Kistler et al., 2017). It is well known that temperature is another major factor influencing DNA preservation. Accordingly, the majority of ancient DNA studies have involved material from cold or temperate environments (Seersholm et al., 2018; Willerslev et al., 2014). With this in mind, it is reasonable to assume that DNA in the P.W. Lund's collection is heavily degraded. In particular, the first 15 years of storage in the warm and humid conditions of his house in Lagoa Santa could have been detrimental to the molecular preservation of the collection.

Bulk bone metabarcoding (BBM) is a recently developed ancient DNA (aDNA) method that allows for a fast and efficient species identification of small fragmentary bones from archeological and paleontological excavations (Murray et al., 2013) (Figure 1c). BBM has been applied in various settings across the globe, ranging from cave assemblages in temperate climates (Grealy et al., 2015; Murray et al., 2013) to tropical fish middens (Grealy et al., 2016). The approach utilizes the vast amounts of nondiagnostic bone fragments collected from bone assemblages. By grinding up and collectively analyzing 25–100 small bone samples, a high‐resolution picture of the species composition in faunal assemblages can be generated. One of the advantages of BBM is that important new biological information can be obtained from small “scraps” of fragmentary, unidentifiable bones, which are typically considered of lower value compared with well‐preserved subfossils. Furthermore, this genetic method allows for an analysis of within‐species genetic variation, which can be used to address questions of past demographic changes within a given population of animals (Haouchar et al., 2014; Seersholm et al., 2018).

In this study, we aim to test whether the bulk bone metabarcoding approach can be applied to obtain new information from the extremely large number of small, fragmented, and unidentified bones stored in the Lund collection. We tested this methodology on four batches of samples containing 25 bones each, excavated from the cave Lapa da Escrivania by Lund.

## 2 RESULTS

**FIGURE 2:**
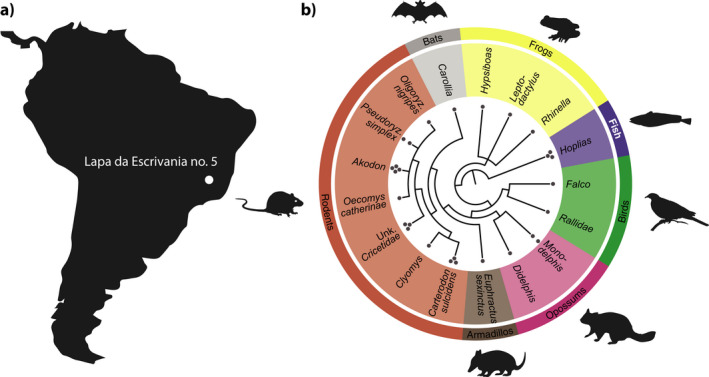
Overview of results. (a) Location of the cave “Lapa da Escrivania no. 5” from which the samples were excavated. (b) Dendrogram of genetic diversity (lowest taxonomic nodes) identified through bulk bone metabarcoding. Black dots at each taxonomic node denote the number of samples in which each taxon was identified. The dendrogram was generated using the standard NCBI taxonomy database (Federhen, 2012) (see Methods)

We collected four samples of ~25 small bones each from P.W. Lund's collection (Figure 1a and Table S1). The samples were collected from a part of the collection consisting of 30 boxes of small animal bones excavated from the cave Lapa de Escrivania no. 5. This part of the collection consists of owl regurgitation mixed with some bone fragments from larger animals (Figure 2). The samples were analyzed with bulk bone metabarcoding (Figure 1c) using four PCR assays: two shorter assays (89–115 bp (Seersholm et al., 2018)) targeting broadly (vertebrates and mammals, respectively), and two longer assays (198–235 bp (Seersholm et al., 2018)) targeting narrower taxonomic groups (fish and birds, respectively; Table S2). This strategy was elected to cover all major groups of vertebrates while ensuring high taxonomic resolution. In agreement with the relatively short size of most damaged ancient DNA (Allentoft et al., 2012), we found that the longer assays only worked in one out of eight reactions, while all reactions were amplified for the two short assays (Table S3).

In total, next‐generation sequencing yielded 323,739 single‐end DNA reads after filtering (35,971 ± 13,266 raw reads per sample per assay, mean ± SD), corresponding to 92 ASVs (amplicon sequence variants; Table S3). Of these, 51 ASVs could be confidently assigned to a taxon. Overall, the analysis yielded 21 different vertebrate taxa from 11 families (Table 1 and Table S4). We find a high abundance of small species with fragile bones: The largest group of species is rodents (10 taxa), followed by frogs (three taxa). However, we also find DNA evidence of larger animals, such as the six‐banded armadillo (*Euphractus sexcinctus*) and a falcon (*Falco* sp.).

**TABLE 1:** Taxa detected from bulk bone metabarcoding compared with the rest of Lund's collection

Taxon	common name	Presence in Lund's collection Recent/Subfossil (R/S)	Presence of taxon in region within last 20 years
*Hoplias* sp.	Trahiras	R,S	Yes
*Rhinella*	Beaked toads	R	No
*Hypsiboas* sp.	Gladiator frogs	R	Likely
*Leptodactylus* sp.	White‐lipped frogs	R	Yes
Neognathae	—	R,S	Yes
*Falco* sp.	Falcon	R,S	Yes
Rallidae	Rail	R,S	Yes
*Didelphinae*	Opossums	R,S	Yes
*Didelphis*	American opossums	R,S	Yes
*Monodelphis* sp.	Short‐tailed opossum	R,S	Yes
*Carterodon sulcidens*	Owl's spiny rat	R,S	Yes
*Clyomys* sp.	—	R,S	Yes
Unknown Cricetidae	—	R,S	?
*Sigmodontinae*	—	R,S	Yes
*Akodon* sp.	Grass mouse	R,S	Likely
*Oecomys catherinae*	Atlantic Forest oecomys	—	No
*Oligoryzomys* sp.	—	—	Likely
*Oligoryzomys nigripes*	Black‐footed pygmy rice rat	—	No
*Pseudoryzomys* sp.	—	R,S	No
*Pseudoryzomys simplex*	Brazilian false rat	R,S	No
*Carollia* sp.	Short‐tailed fruit bats	R,S	No
*Euphractus sexcinctus*	six‐banded armadillo	R,F	Yes

Note

Only taxa detected at a taxonomic resolution at family level or below are shown, and contaminants were excluded (see Table S4 for the full data table). The column “Presence in Lund's collection” describes whether a given taxon was identified in Lund's collection. Recent: present around Lagoa Santa at the same time as P.W. Lund (1835–1880). Subfossil: subfossil material of the taxon present in the Lund collection. Data in the right‐most column represent the taxon presence in the Lagoa Santa region (from year 2000‐) and are based on GBIF and museum records at NHMD.

A total of six negative controls were included in the sample processing workflow (two grinding blanks, two extractions blanks, and two PCR blanks). Two contaminant ASVs were detected from the negative controls that amplified: one from chicken (*Gallus gallus*) and one from human being (*Homo sapiens*). Additionally, human DNA was detected in all test samples, while another common contaminant (cattle; *Bos* sp.) was detected in sample 3, but not in the negative controls. As all of these taxa are routinely identified as common laboratory contaminants (Haile et al., 2009; Leonard et al., 2007; Seersholm et al., 2016), these were labeled as contamination and removed from downstream analyses.

To compare bulk bone metabarcoding with a more traditional approach, we also identified the bones morphologically (Table S5). In agreement with the genetic record, the morphological identifications found rodents to be abundant in all four samples. Furthermore, although less abundant than rodents, amphibians were detected morphologically in all samples. Amphibians were also detected in the DNA record (beaked toads, *Rhinella*; gladiator frogs, *Hypsiboas;* and white‐lipped frogs, *Leptodactylus* sp.), albeit only in samples 3 and 4. Similarly, birds were detected in all samples morphologically, but the two bird species identified by DNA (Falcon, *Falco* sp.; and rail, Rallidae) were only detected in samples 1 and 4. Conversely, for mammal species other than rodents, the DNA‐based method was able to detect a wider diversity than that reported morphologically. Three species of larger mammals were detected genetically (American opossums, *Didelphis* sp.; short‐tailed opossum, *Monodelphis* sp.; and six‐banded armadillo, *E. sexcinctus*), along with one species of bat (short‐tailed fruit bats, *Carollia* sp.). Morphologically, only one unidentified large mammal species was detected in sample 4.

While the overall faunal diversity detected using both morphology and DNA is similar, there are obvious differences between the two approaches. As described above, bird DNA is absent from samples 1 and 2, despite the detection of bird bones morphologically in these samples. This discrepancy could be explained by poor primer binding to the bird DNA or the absence of relevant reference sequences in public genetic databases. Furthermore, the detection of trahiras (*Hoplias* sp.) in three of four samples is surprising, as no fish bones were noticed among the bulk bone samples. The presence of trahira DNA in the absence of clearly identifiable fish bones could, in principle, stem from laboratory contamination. However, this is unlikely given the measures taken to control for contamination in this study (see Methods). We do not find trahira DNA in any of the negative controls, and trahira has not before been described as a common laboratory contaminant in the literature. As the *Hoplias* genus is endemic to South America, it is more likely that this genetic signature represents either (a) ancient remains from the cave or (b) potential contamination during the excavation 150 years ago. As for the first possibility, the fish DNA could potentially originate from highly fragmented bones or “bone dust,” which would be almost impossible to identify morphologically. Alternatively, trahira DNA could have been deposited in the cave sediments (and on nonfish subfossils) via feces or blood/tissue remains from the activity of fish‐eating predators. Indeed, trahiras are present in the area around Lagoa Santa, and it is plausible that it was brought into the cave by a predator (e.g., *Falco* sp.). As for the second possibility, trahira DNA could have been deposited on the surface of the bones during the excavation. If the subfossils were washed in water from a stream nearby, it is possible that trace amounts of trahira DNA in the water contaminated the samples. This scenario is unlikely, however, given the low concentration of fish DNA generally detected in water samples and the age of the samples.

Between 6 and 11 different taxa were identified in each sample. Compared with other bulk bone metabarcoding studies, this is surprisingly high, in particular because of the small sample size (25 bone fragments). In addition, even sample four, included due to its appearance as a poorly preserved sample with very porous and fragmented bones, yielded nine different taxa. This indicates that this part of the Lund collection has excellent DNA preservation and demonstrates that future more comprehensive aDNA studies on this part of the collection are likely to be successful.

Even though some taxa detected by DNA were not identified morphologically in the same samples, most of the taxa detected have been identified before in the P.W. Lund collection as a whole (Table 1, Table S6, and Table S7). While some of the species were found in both the subfossil and recent sections of the collection, others were only found in the recent collection (*Rhinella* sp., *Hypsiboas* sp., and *Leptodactylus* sp.). However, it is important to note that several of the species recorded in P.W. Lund's collection were identified more than 100 years ago and require review by current experts. In particular need are Aves, Rodentia, and Amphibia. This is exemplified by the two species detected by BBM that have not been registered before in the collection using classic morphology: *Oecomys catherinae* and *Oligoryzomys nigripes*. Both of these species are notoriously difficult to identify, and related taxa have been identified in the collection. It is not unlikely that these two species could be identified morphologically in the collection if the material was reviewed by a contemporary expert on Brazilian rodents.

## 3 DISCUSSION

With a long and turbulent history, the P.W. Lund collection has been stored under far from ideal conditions since the bones were unearthed 180 years ago. Despite the vast size and significance of the collection, the attempts of DNA extraction have only been successful on human remains from the petrous bones of two individuals (Moreno‐Mayar et al., 2018). The first DNA efforts, based on tooth samples, were initially fruitless, but after switching to petrous bone, the endogenous human DNA content was sufficiently high for genomic sequencing (Moreno‐Mayar et al., 2018). However, the small animal bones that constitute the vast majority of the collection have never been tested for DNA preservation. By comparing bulk bone metabarcoding results from two mitochondrial regions (*12S* and *16S*), we demonstrate DNA preservation in four samples of 25 small bone fragments from P.W. Lund's collection. Even sample 4, which was selected because of its physical appearance as a poorly preserved sample, yielded positive results. These findings demonstrate that even the smallest ancient bones from the collection yield endogenous DNA, despite its age and its turbulent history, involving periods of highly unfavorable preservation conditions.

Two of the taxa identified by DNA (*O. catherinae* and *O. nigripes*) are new identifications, which have not been registered in Lund's collection before. This finding illustrates that new species identifications are likely to be made if the BBM method is applied across a much larger sample of the collection. The application of BBM is particularly promising in the breccias found in the P.W. Lund collection, and in owl regurgitation samples similar to the samples tested here. Prior to our analyses, none of these samples had been formally analyzed. The owl regurgitation consists of approximately two million small bones and bone fragments, primarily from small mammals, birds, amphibians, and fish. The breccias, numbering more than 1,300 pieces, consist of a limestone matrix wherein fragments of subfossil bones remain. These subfossils include several extinct species of mammals and *H. sapiens*. For these parts of the collection, BBM could prove an efficient and cost‐effective approach to provide an overview of the fauna present. Furthermore, the combination of metabarcoding and shotgun sequencing methods on Lund's collection could possibly provide whole‐genome sequence data from species of interest while preserving cost efficiency. By focusing shotgun sequencing efforts on samples where the species of interest have already been detected with bulk bone metabarcoding, sequencing cost can be reduced dramatically. In particular, megafaunal species such as saber‐toothed cat (*S. populator*) and glyptodonts (*Glyptodon* sp.), from which well‐preserved morphologically identifiable bone fragments are rare, would be ideal candidates for such analyses.

As outlined above, the potential of applying large‐scale genetic studies across the Lund collection is considerable, but a series of limitations still remain to be overcome in order to take full advantage of the samples. Most of these limitations relate to how the samples were collected. For example, Lund's excavation protocol did not involve the sorting of subfossils based on the strata from which they were excavated. As a result, Lund's collection is stored in boxes with very limited metadata and with no information on excavation depth. Hence, it could prove costly to date bones for DNA analysis, as essentially every bone analyzed would have to be dated separately. Another challenge is caused by the age of many of the taxonomic identifications performed on the collection, which were conducted over 100 years ago. Since significant taxonomic revisions have been carried out on South American fauna, many of these identifications are outdated. Lastly, identifying the original sources of the DNA detected in this study (and environmental DNA studies in general) can also constitute a challenge. The detection of trahira (*Hoplias* sp.) DNA in the absence of morphologically identifiable fish remains, for example, suggests that other sources than endogenous DNA from the bones could also have contributed. It is possible that the fish DNA stems from feces or leftovers from a predator's meal or bone dust as discussed above. However, given the high level of agreement between the species identified in our data and the morphological analysis of the collection as a whole, we do not expect such deposition of exogenous DNA (i.e., contamination) to be a major concern.

Museum collections around the world harbor countless specimens with relevance for future genetic studies. The fact that one of the oldest collections with the most tumultuous histories still yields endogenous DNA suggests that DNA could be retrieved from many similar 19th‐century collections, particularly those of Holocene age. At the Natural History Museum in Copenhagen for example, another less famous 19th‐century South American subfossil collection is stored—that of Dr. Valdemar Lausen. With the combined genetic insights from both the P.W. Lund collection of Brazilian subfossils and Dr. Valdemar Lausen's collections from Argentina, the biodiversity of South American paleontology could take a significant leap forward.

## 4 METHODS

Four samples of 25 bones each were collected from the Lund collection at the Natural History Museum of Denmark in January 2017. The samples originate from P.W. Lund's excavation at the cave *Lapa da Escrivania no. 5* in Lagoa Santa, Minas Gerais State, Brazil, and were collected from box 1–4, respectively (Table S1). Samples were processed at the TRACE (Trace Research Advanced Clean Environment) aDNA facility at Curtin University, Western Australia, following strict ancient DNA guidelines (Willerslev & Cooper, 2005): All handling of the samples was carried out wearing gloves, facemask, and a full bodysuit, and each step of sampling preparation (bone grinding, DNA extraction, and PCR setup) was performed in separate laboratories within the clean laboratory facility. Furthermore, all instruments and surfaces used were cleaned with a 10% bleach solution, followed by a 70% ethanol solution. For bone grinding, subfossil fragments were subsampled to ensure that each bone was of roughly equal size (~100 mg). Next, the subsampled fragments were ground using a Retsch PM 200 Planetary Ball Mill at 400 rpm until pulverized. To control for contamination at the bone grinding step, two grinding blanks were included in the workflow. The grinding blanks consisted of 15 ml of ultrapure water that was added to the clean grinding pod and run on the ball mill. After grinding, the water was concentrated to 500 µl on an Amicon® Ultra‐4 Centrifugal Filter (Millipore) and analyzed like the test samples. Ancient DNA was extracted using a modified version of the extraction protocol described by Dabney et al. (2013), including two nontemplate extraction blanks. After DNA extraction, samples were analyzed using four metabarcoding assays in which barcode regions of two mitochondrial genes (*12S* and *16S rRNA* gene) were amplified with primers targeting vertebrates (12SV5; Riaz et al., 2011), mammals (Mam16S; Taylor, 1996), fish (Fish16S; Deagle et al., 2007), and birds (12SAH; Cooper, 1994). These metabarcoding primers were fused with Illumina sequencing adapters and a 6‐ to 8‐bp index to identify each sample. Hence, amplification with gene‐specific primers and library preparation was achieved in a single amplification step. Two nontemplate PCR blanks were added to the workflow to control for contamination during the PCR. Lastly, amplified PCR products were sequenced on the Illumina MiSeq sequencing platform in single‐end configuration for 325 cycles on a standard flow cell using V2 chemistry.

After DNA sequencing, reads were demultiplexed and filtered using a custom‐made OBItools pipeline (https://pythonhosted.org/OBITools/welcome.html#installing‐the‐obitools). First, raw fastq files were demultiplexed based on the 6‐ to 8‐bp index tag and the gene‐specific primers using *ngsfilter* from OBItools. Next, reads were dereplicated with obiuniq and filtered with obigrep set to only retain unique reads longer than 80 bp and represented by more than 10 reads in a sample. To filter out artifacts from PCR and sequencing errors, we applied three steps of denoising: Obiclean (r 0.2 ‐d 2 ‐H) and Sumaclust, collapsing clusters at 95% and 93% with abundance thresholds of 50% and 1%, respectively, in a sample‐wise manner. Lastly, chimeric sequences were removed using vsearch (vsearch ‐‐uchime_denovo). Next, ASVs were queried against the NCBI nt database using blast (Altschul et al., 1990) and assigned to the taxonomic nodes of the best hit(s) using the script blast_getLCA.py (https://github.com/frederikseersholm/blast_getLCA; Seersholm et al., 2016, 2018) Lastly, raw taxonomic assignments were scrutinized and compared with records of taxa currently present around Lagoa Santa. For example, if relevant species were missing from the database, hits to closely related species were dropped to genus level.

The dendrogram in Figure 2 was generated based on the NCBI taxonomy of the species identified with BBM used the script create_tree_from_curated_list.py (https://github.com/frederikseersholm/blast_getLCA ).

## CONFLICT OF INTEREST

The authors declare no competing interests.

## AUTHOR CONTRIBUTIONS

**Frederik Seersholm:** Data curation (lead); formal analysis (equal); investigation (lead); methodology (lead); project administration (lead); software (lead); visualization (lead); writing – original draft (equal); writing – review and editing (equal). **Kasper Lykke Hansen:** Formal analysis (equal); investigation (equal); methodology (equal); writing – original draft (equal); writing – review and editing (equal). **Matthew Heydenrych:** Conceptualization (supporting); formal analysis (equal); investigation (equal); writing – review and editing (equal). **Anders J. Hansen:** Conceptualization (equal); funding acquisition (equal); supervision (equal); writing – review and editing (equal). **Michael Bunce:** Conceptualization (equal); funding acquisition (equal); supervision (equal); writing – review and editing (equal). **Morten E. Allentoft:** Conceptualization (equal); formal analysis (equal); funding acquisition (equal); project administration (equal); supervision (equal); writing – original draft (equal); writing – review and editing (equal).

## Supporting information

Appendix S1

## Data Availability

Fastq files for all DNA sequencing data reported in this paper were deposited in the European Nucleotide Archive under study accession number PRJEB40614.
