# Supplementary material for: Ancient DNA preserved in small bone fragments from the P.W. Lund collection
Source: Ecol Evol. 2021 Feb 5;11(5):2064–71. doi: 10.1002/ece3.7162 (PMC7920760; doi:10.1002/ece3.7162)
Supplement: Supplementary file 1 — Appendix S1 [file ECE3-11-2064-s001.docx]

**Appendix S1**

Excellent DNA Preservation in Ancient Bones from the P.W. Lund Collection

Seersholm et al.

**Table S1.** **Bulk bone samples from Lund’s collection.** DNA preservation was visually inspected and sorted into two categories: Average and Poor. Average preservation is defined as subfossils with a smooth and hard exterior, and a general appearance comparable to the rest of the collection. Poor preservation is defined as brittle and relatively light subfossils with clear indications of bone degradation.

| Sample name | n  bones |  | Bone  powder sampled | Description | Preser-vation |
| --- | --- | --- | --- | --- | --- |
| Sample 1 | 25 |  | 161mg | Escrivania no.5. lagoa Santa, Brazil. "Box 1" | Average |
| Sample 2 | 25 |  | 119 mg | Escrivania no.5. lagoa Santa, Brazil. "Box 2" | Average |
| Sample 3 | 25 |  | 99 mg | Escrivania no.5. lagoa Santa, Brazil. "Box 3" | Average |
| Sample 4 | 26 |  | 111 mg | Escrivania no.5. lagoa Santa, Brazil. "Box 4" | Poor |

**Table S2. Barcoding assays.** Amplicon sizes presented are from this study.

| *Amplicon* | *Forward Primer*  *5’ – 3’* | *Reverse Primer*  *5’ – 3’* | *Annealing Temperature* | *Reference* |
| --- | --- | --- | --- | --- |
| 12SV5 | TAGAACAGGCTCCTCTAG | TTAGATACCCCACTATGC | 57°C | Riaz et al. (2011) ^1^ |
| Mam16S | CGGTTGGGGTGACCTCGGA | GCTGTTATCCCTAGGGTAACT | 57°C | Taylor (1996) ^2^ |
| Fish16S | GACCCTATGGAGCTTTAGAC | CGCTGTTATCCCTADRGTAACT | 54°C | Deagle et al. (2007)^3^ |
| 12SAH | CTGGGATTAGATACCCCACTAT | CCTTGACCTGTCTTGTTAGC | 57°C | Cooper (1994)^4^ |

**Table S3. Sequencing counts of raw (unfiltered) reads, filtered reads, and unique reads.** Almost all reads amplified by the two longer assays (12Sah and Fish16S) represents non-specific amplification of the gene specific primers and the sequencing primers without the expected DNA insert between them. These were removed in the filtering pipeline due to the length cut off of 80bp. EB: Extraction blank, GB: grinding blank, PB: PCR blank (non-template control).

| Sample name | PCR assay | Raw  count | Filtered  count | Unique reads |
| --- | --- | --- | --- | --- |
| Sample 1 | 12Sah | 13631 | 0 | 0 |
| Sample 1 | 12Sv5 | 75319 | 39859 | 7 |
| Sample 1 | Fish16S | 8923 | 0 | 0 |
| Sample 1 | Mam16S | 70297 | 39498 | 8 |
| Sample 2 | 12Sv5 | 93944 | 49199 | 15 |
| Sample 2 | Fish16S | 5626 | 0 | 0 |
| Sample 2 | Mam16S | 65116 | 42297 | 12 |
| Sample 3 | 12Sv5 | 66998 | 38744 | 12 |
| Sample 3 | Fish16S | 9425 | 2452 | 2 |
| Sample 3 | Mam16S | 58087 | 37794 | 17 |
| Sample 4 | 12Sah | 2 | 0 | 0 |
| Sample 4 | 12Sv5 | 65490 | 39805 | 17 |
| Sample 4 | Fish16S | 4381 | 0 | 0 |
| Sample 4 | Mam16S | 69382 | 46342 | 10 |
| EB1 | 12Sah | *no amplification* | | |
| EB1 | 12Sv5 | *no amplification* | | |
| EB1 | Fish16S | 2119 | 0 | 0 |
| EB1 | Mam16S | *no amplification* | | |
| EB2 | 12Sah | *no amplification* | | |
| GB1 | 12Sah | *no amplification* | | |
| GB1 | 12Sv5 | 90125 | 63790 | 1 |
| GB1 | Fish16S | 1377 | 0 | 0 |
| GB1 | Mam16S | 81 | 0 | 0 |
| GB2 | 12Sah | 229 | 168 | 1 |
| PB1 | 12Sah | *no amplification* | | |
| PB1 | 12Sv5 | 459 | 0 | 0 |
| PB1 | Fish16S | 1803 | 0 | 0 |
| PB1 | Mam16S | *no amplification* | | |
| PB2 | 12Sah | 1 | 0 | 0 |

**Table S4.** Taxa detected from Bulk Bone Metabarcoding on four samples from P.W. Lund’s collection. Numbers indicate the number of metabarcoding assays each taxon was detected with. S1-4 represents the four bulk bone samples analysed and negative controls represents the data from grinding blanks, extraction blanks and PCR blanks (non-template controls) outlined in Table S3. *Common laboratory contaminants.

| Taxon | common name | S1 | S2 | S3 | S4 | Neg. controls |
| --- | --- | --- | --- | --- | --- | --- |
| *Hoplias sp.* | Trahiras | 1 | 1 | 1 | - | - |
| *Rhinella* | Beaked toads | - | - | - | 1 | - |
| *Hypsiboas sp.* | Gladiator frogs | - | - | 1 | - | - |
| *Leptodactylus sp.* | White-lipped frogs | - | - | 3 | - | - |
| Neognathae | - | - | - | - | 1 | - |
| *Falco sp.* | Falcon | - | - | - | 1 | - |
| Rallidae | Rail | 1 | - | - | - | - |
| *Didelphinae* | Opossums | - | - | - | 1 | - |
| *Didelphis* | American opossums | - | - | - | 1 | - |
| *Monodelphis sp.* | Short tailed opossum | - | 2 | - | 2 | - |
| *Carterodon sulcidens* | Owl's spiny rat | 2 | 2 | 2 | - | - |
| *Clyomys sp.* | - | - | - | - | 2 | - |
| Unknown Cricetidae | - | - | 1 | 1 | 1 | - |
| *Sigmodontinae* | - | 1 | - | 1 | - | - |
| *Akodon sp.* | Grass mouse | 1 | 1 | 1 | 1 | - |
| *Oecomys catherinae* | Atlantic Forest oecomys | - | - | 1 | - | - |
| *Oligoryzomys sp.* | - | - | - | 1 | - | - |
| *Oligoryzomys nigripes* | Black-footed pygmy rice rat | - | - | 1 | - | - |
| *Pseudoryzomys sp.* | - | - | 1 | - | - | - |
| *Pseudoryzomys simplex* | Brazilian false rat | - | 1 | - | 1 | - |
| *Carollia sp.* | Short-tailed fruit bats | - | - | 1 | - | - |
| *Euphractus sexcinctus* | six-banded armadillo | 1 | - | - | - | - |
| *Bos* sp.* | Cattle | - | - | 1 | - | - |
| *Gallus gallus** | Chicken | - | - | - | - | 1 |
| *Homo sapiens** | Human | 2 | 1 | 2 | 2 | 1 |

**Table S5.** Brief morphological characterisation of sampled bones.

|  | Sample 1 | | Sample 2 | | Sample 3 | | Sample 4 | |
| --- | --- | --- | --- | --- | --- | --- | --- | --- |
|  | Indivi-duals | Species | Indivi-duals | Species | Indivi-duals | Species | Indivi-duals | Species |
| Mammals | 12 | 3 | 11 | 4 | 7 | 4 | 4 | 4 |
| Birds | 4 | 3 | 3 | 2 | 3 | 1 | 9 | 3 |
| Herpetofauna | 2 | 2 | 3 | 2 | 3 | 3 | 2 | ? |

**Table S6.** Taxa represented in the P.W. Lund collection based on morphology. P.W. Lund collected both fossil and recent specimens during his most scientifically productive period in Brazil (1835-1845).

| Taxon | common name | Fossil  Collection | Recent  Collection | Taxon in ZM |  |
| --- | --- | --- | --- | --- | --- |
| *Hoplias sp.* | Trahiras | X | X | *H. malabaricus* (Bloch, 1794) |  |
| *Rhinella sp.* | Beaked toads | - | X | *R. marina* (Linnaeus, 1758) |  |
| *Hypsiboas sp.* | Gladiator frogs | - | X | *H. boans* (Linnaeus, 1758)  *H. lundii* (Burmeister, 1856)  *H. pardalis* (Spix, 1824) |  |
| *Leptodactylus sp.* | White-lipped frogs | - | X | *L. fuscus* (Schneider, 1799)  *L. labyrinthicus* (Spix, 1824)  *L. mystacinus* (Burmeister, 1861) |  |
| *Falco sp.* | Falcon | X | X | *F. sparverius* Linnaeus, 1758  *F. femoralis* Temminck, 1822 |  |
| Rallidae | Rail | X | X | Several species |  |
| *Didelphinae* | Opossums | X | X | Several species |  |
| *Didelphis sp.* | American opossums | X | X | Several species |  |
| *Monodelphis sp.* | Short tailed opossum | X | X | *M. domestica* (Wagner 1842) |  |
| *Carterodon sulcidens* | Owl's spiny rat | X | X | *Carterodon sulcidens* (Lund, 1841) |  |
| *Clyomys sp.* | - | X | X | C. laticeps (Thomas 1909) |  |
| Unknown Cricetidae | - | X | X | Several species |  |
| *Sigmodontinae* | - | X | X | Several species |  |
| *Akodon sp.* | Grass mouse | X | X | *Thalopomys lasiotis* Thomas, 1916 |  |
| *Oecomys catherinae* | Atlantic Forest oecomys | - | - | Not found |  |
| *Oligoryzomys sp.* | - | - | - | Not found |  |
| *Oligoryzomys nigripes* | Black-footed pygmy rice rat | - | - | Not found |  |
| *Pseudoryzomys sp.* | Note only 1 species in genus | X | X | *Pseudoryzomys simplex* (Winge 1887) |  |
| *Pseudoryzomys simplex* | Brazilian false rat | X | X | *Pseudoryzomys simplex* (Winge 1887) |  |
| *Carollia sp.* | Short-tailed fruit bats | X | X | *C. brevicaudata* Wied-Neuwied, 1821 |  |
| *Euphractus sexcinctus* | six-banded armadillo | X | X | *Euphractus sexcinctus* (Linnaeus 1758) |  |

**Table S7.** Taxa presence in Lagoa Santa region (from year 2000-). Based on GBIF & personal correspondence

| Taxon | common name | Taxon name in ZM | Presence of ZM taxon in region within last 20 years |
| --- | --- | --- | --- |
| *Hoplias sp.* | Trahiras | *H. malabaricus* (Bloch, 1794) | Yes |
| *Rhinella sp.* | Beaked toads | *R. marina* (Linnaeus, 1758) | No |
| *Hypsiboas sp.* | Gladiator frogs | *H. boans* (Linnaeus, 1758)  *H. lundii* (Burmeister, 1856)  *H. pardalis* (Spix, 1824) | No  Likely  Likely |
| *Leptodactylus sp.* | White-lipped frogs | *L. fuscus* (Schneider, 1799)  *L. labyrinthicus* (Spix, 1824)  *L. mystacinus* (Burmeister, 1861) | Yes  Yes  Yes |
| *Falco sp.* | Falcon | *F. sparverius* Linnaeus, 1758  *F. femoralis* Temminck, 1822 | Yes  Yes |
| Rallidae | Rail | Several species | yes |
| *Didelphinae* | Opossums | Several species | Yes |
| *Didelphis sp.* | American opossums | Several species | Yes |
| *Monodelphis sp.* | Short tailed opossum | *M. domestica* (Wagner 1842) | No |
| *Carterodon sulcidens* | Owl's spiny rat | *Carterodon sulcidens* (Lund, 1841) | Yes |
| *Clyomys sp.* | - | *C. laticeps* (Thomas 1909) | Yes |
| Unknown Cricetidae | - | Several species | ? |
| *Sigmodontinae* | - | Several species | Yes |
| *Akodon sp.* | Grass mouse | *Thalopomys lasiotis* Thomas, 1916 | Likely |
| *Oecomys catherinae* | Atlantic Forest oecomys | Not found | (No) |
| *Oligoryzomys sp.* | - | Not found | (Likely) |
| *Oligoryzomys nigripes* | Black-footed pygmy rice rat | Not found | (No) |
| *Pseudoryzomys sp.* | Note only 1 species in genus | *Pseudoryzomys simplex* (Winge 1887) | No |
| *Pseudoryzomys simplex* | Brazilian false rat | *Pseudoryzomys simplex* (Winge 1887) | No |
| *Carollia sp.* | Short-tailed fruit bats | *C. brevicaudata* Wied-Neuwied, 1821 | No |
| *Euphractus sexcinctus* | six-banded armadillo | *Euphractus sexcinctus* (Linnaeus 1758) | Yes |

**Supplementary Notes on morphology**

**Sample 1:** “Box 1” (P21/2017KMG): Mainly mammalian (NISP: 19), dominated by rodents, and representing at least 12 individuals from at least 3 different species. 3 maybe 4 bird bones from at least 2-3 individuals and possibly 3 different species. Reptiles and amphibians are also present with 2 bones representing as many individuals. The total weight comes to about 3.3 grams.

**Sample 2:** “Box 2” (P22/2017KMG): Mainly mammalian (NISP: 19), dominated by rodents, and representing at least 11 individuals from at least 4 different species. 3 bird bones from at least 2 individuals representing at least 2 different species. Reptiles and amphibians are also present with 3 bones representing at least 2 individuals. The total weight comes to about 2.5 grams.

**Sample 3:** “Box 3” (P23/2017KMG): Mainly mammalian (NISP: 18), dominated by rodents, and representing at least 7 individuals from at least 4 different species. 3 bird bones from at least 1 individual representing at least 1 species. Reptiles and amphibians are also present with 2, maybe 3, bones representing at least 2, maybe 3, individuals. The total weight comes to about 2.4 grams.

**Sample 4:** “Box 4” (P24/2017KMG): Mainly mammalian (NISP: approx. 14), dominated by rodents but more varied than the former selections and contains larger species of mammals and representing more than 4 different species. Approx. 9 bird bones from at least 3 different species. Some quite large. Reptiles and amphibians are also present with 2 bones. The total weight comes to about 4.5 grams.
